# How to become a strategic purchaser of rehabilitation services

**DOI:** 10.2471/BLT.21.287499

**Published:** 2022-10-03

**Authors:** Tamara Chikhradze, Emma L Brainerd, Adeel Ishtiaq, Reva Alperson

**Affiliations:** a Results for Development, Suite 700, 1111 19th Street, Washington, District of Columbia, 20036, United States of America.

## Abstract

Rehabilitative care is often overlooked and underfunded despite being a key component of universal health coverage, and now faces further neglect due to indirect impacts of the coronavirus disease 2019 pandemic. Policy-makers can leverage strategic purchasing approaches to make the most of available funds and maximize health gains. To implement more strategic purchasing of rehabilitation, health planners must: (i) develop and prioritize evidence-based rehabilitation service packages; (ii) use fit-for-purpose contracting and provider payment mechanisms to incentivize quality and efficient service delivery; and (iii) strengthen stewardship. This paper examines these three policy priorities by analysing their associated processes, actors and resources based on country experiences. Policy-makers will likely face several obstacles in operationalizing these policy priorities, including: inadequate accountability and coordination among sectors; limited data and research; undefined and non-standardized rehabilitation services, costs and outcomes; and inadequate availability of rehabilitative care. To overcome challenges and institute optimal strategic purchasing practices for rehabilitation, we recommend that policy-makers strengthen health sector stewardship and establish a framework for multisectoral collaboration, invest in data and research and make use of available experience from high-income settings, while creating a body of evidence from low- and middle-income settings.

## Résumé

Les soins de réadaptation sont souvent négligés et sous-financés malgré la place essentielle qu'ils occupent dans la couverture sanitaire universelle. Aujourd'hui, ils risquent même d'être relégués au second plan à la suite des conséquences indirectes de la pandémie de maladie à coronavirus 2019. Les responsables politiques peuvent néanmoins adopter des méthodes d'achat stratégiques afin de tirer le meilleur parti des fonds disponibles et de maximiser les bénéfices pour la santé. Pour ce faire, les planificateurs sanitaires doivent: (i) développer et privilégier les programmes de réadaptation étayés par des faits; (ii) utiliser des contrats adéquats et des mécanismes de paiement des fournisseurs qui mettent en valeur la qualité et l'efficacité des prestations de services; et enfin, (iii) renforcer les activités de gestion. Le présent document se penche sur ces trois priorités politiques en analysant les processus, acteurs et ressources qui y sont associés dans différents pays. Les décideurs seront probablement confrontés à de nombreux obstacles lors de la mise en œuvre de telles politiques: un manque de responsabilisation et de coordination entre secteurs; des recherches et données limitées; une absence de normalisation et de définition claire des services, coûts et résultats; et des soins de réadaptation en pénurie. Pour relever ces défis et instaurer des pratiques d'achat stratégiques optimales en matière de réadaptation, nous leur conseillons de renforcer la gestion du secteur de la santé et d'établir un cadre de collaboration multisectorielle, d'investir dans la recherche et la collecte de données, et de s'inspirer des expériences vécues dans les régions à revenu élevé tout en récoltant un ensemble de preuves dans les régions à revenu faible et intermédiaire.

## Resumen

La atención de rehabilitación suele pasar desapercibida y carecer de fondos a pesar de ser un componente clave de la cobertura sanitaria universal, y ahora se enfrenta a una mayor desatención debido a las repercusiones indirectas de la pandemia de la enfermedad por coronavirus de 2019. Los responsables de formular las políticas pueden aprovechar los enfoques de adquisición estratégica para sacar el máximo provecho de los fondos disponibles y maximizar los beneficios para la salud. Para aplicar una adquisición más estratégica en materia de rehabilitación, los planificadores sanitarios deben (i) desarrollar y priorizar paquetes de servicios de rehabilitación a partir de la evidencia; (ii) utilizar mecanismos de contratación y pago a proveedores adecuados para incentivar la calidad y la prestación eficiente de los servicios; y (iii) fortalecer la administración. El presente documento estudia estas tres prioridades políticas mediante el análisis de sus procesos, actores y recursos asociados, basándose en las experiencias de los países. Es probable que los responsables de formular las políticas se enfrenten a varios obstáculos a la hora de poner en práctica estas prioridades políticas, entre los que se incluyen: una responsabilidad y coordinación inadecuadas entre sectores; la limitación de los datos y la investigación; la falta de definición y estandarización de los servicios, los costes y los resultados de la rehabilitación; y la insuficiente disponibilidad de la atención de rehabilitación. Para superar los desafíos e instituir prácticas estratégicas óptimas de adquisición en materia de rehabilitación, se recomienda que los responsables de formular las políticas fortalezcan la administración del sector sanitario y establezcan un marco de colaboración multisectorial, inviertan en datos e investigación y aprovechen la experiencia disponible en entornos de ingresos altos, al tiempo que crean un conjunto de evidencias procedentes de entornos de ingresos bajos y medios.

## ملخص

غالبًا ما يتم تجاهل الرعاية التأهيلية ولا يتم تمويلها بدرجة كافية على الرغم من أنها مكون رئيسي في التغطية الصحية العالمية، ونحن نواجه الآن إهمالاً إضافيًا بسبب التأثيرات غير المباشرة لجائحة مرض فيروس كورونا الذي ظهر عام 2019. يستطيع مقررو السياسات الاستعانة بنُهج الشراء الاستراتيجي لتحقيق أقصى استفادة من الأموال المتاحة وتعظيم المكاسب الصحية. لشراء خدمات إعادة التأهيل مع التركيز على الجانب الاستراتيجي بدرجة أكبر، يجب على مخططي الرعاية الصحية: (1) تصميم حزم لخدمات إعادة التأهيل القائمة على الأدلة ومنحها الأولوية؛ و(2) استخدام آليات تفي بالغرض فيما يتعلق بالتعاقدات وسداد مستحقات موفري الخدمات بهدف التشجيع على تقديم خدمات فعالة وذات جودة عالية؛ و(3) تعزيز الإدارة الجيدة. تتناول هذه الورقة الأولويات الثلاث للسياسة المذكورة عن طريق تحليل العمليات المرتبطة بها والجهات الفاعلة والموارد مع مراعاة تجارب كل بلد. من المحتمل أن يواجه مقررو السياسات عقبات عديدة في تفعيل أولويات السياسة المشار إليها، ويشمل ذلك: ضعف المساءلة وغياب التنسيق بين القطاعات المختلفة؛ ومحدودية البيانات والأبحاث؛ وخدمات إعادة التأهيل غير المحددة أو غير القياسية وتكاليفها ونتائجها؛ وعدم التوفر الكافي للرعاية التأهيلية. من أجل التغلب على الصعوبات وتأسيس ممارسات مُثلى لشراء خدمات إعادة التأهيل بمنظور استراتيجي، نوصي مقرري السياسات بتعزيز الإدارة الجيدة للقطاع الصحي، ووضع إطار عمل للتعاون بين القطاعات المختلفة، والاستثمار في البيانات والأبحاث والاستفادة من التجارب المتاحة للدول المرتفعة الدخل، مع إنشاء منظومة للأدلة المشتقة من الدول المنخفضة والمتوسطة الدخل.

## 摘要

尽管康复护理已成为全民医疗保险中的重要组成部分,但仍经常被忽视并存在资金不足的问题。由于新型冠状病毒肺炎大流行的间接影响,现在康复护理面临着被进一步忽视的问题。决策制定者可以通过战略采购方法充分利用现有资金并最大限度地提高健康收益。为了实施更具战略性的康复服务采购计划,健康规划者必须:(I) 制定和优先考虑基于循证的综合康复服务;(ii) 使用量身定制的承包商和服务提供商付款机制,以激励高质量和高效的服务交付;以及 (iii) 加强管理工作。本论文基于各国的经验,通过分析各国的相关流程、参与者和资源,对三项政策的优先执行情况进行审查。政策制定者在优先执行这些政策时可能面临一些障碍,包括:部门之间的责任划分和协作不足;有限的数据和研究;未定义和非标准化的康复服务、费用和效果;以及所提供的康复护理服务不足。为了克服这些挑战和为康复服务制定最佳的战略采购实践,我们建议政策制定者加强卫生部门管理,建立多部门协作框架,投资数据开发和研究项目以及利用高收入地区的现有经验,同时在中低收入地区创建大量可用证据。

## Резюме

Несмотря на то что реабилитационная помощь является ключевым компонентом всеобщего охвата услугами здравоохранения, ее часто игнорируют и недостаточно финансируют. В настоящее время наблюдается дальнейшее игнорирование этой проблемы из-за косвенных последствий пандемии коронавирусной инфекции 2019 года. Лица, принимающие решения, могут задействовать стратегические подходы к закупкам, чтобы извлечь максимальную выгоду из имеющихся средств, а также получить максимальный эффект для здоровья человека. Чтобы более эффективно осуществлять стратегические закупки реабилитационных услуг, лица, занимающиеся планированием здравоохранения, должны: (i) разработать пакеты научно обоснованных реабилитационных услуг и определить их приоритетность; (ii) использовать целевые механизмы заключения договоров и оплаты поставщиков для стимулирования качества и эффективности предоставления услуг; (iii) усилить руководство. В данном документе рассматриваются данные политические приоритеты путем анализа связанных с ними процессов, действующих лиц и ресурсов на основе опыта стран. Лица, принимающие стратегические решения, скорее всего, столкнутся с рядом препятствий при реализации этих политических приоритетов, а именно: недостаточной подотчетностью и координацией между секторами, ограниченными данными и исследованиями, неопределенными и нестандартизированными реабилитационными услугами, затратами и результатами; недостаточной доступностью реабилитационной помощи. Чтобы преодолеть трудности и внедрить оптимальные методы стратегических закупок для реабилитации, авторы рекомендуют лицам, принимающим стратегические решения, усилить руководство сектором здравоохранения и создать основу для межсекторального сотрудничества, инвестировать в данные и исследования, а также использовать имеющийся опыт в странах с высоким уровнем дохода, создавая при этом совокупность фактических данных для стран с низким и средним уровнем дохода.

## Introduction

One third of the world's population are living with health conditions that might benefit from rehabilitation services, which improve functioning and are fundamental to enhancing well-being and quality of life.^1^^,^^2^ The coronavirus disease 2019 (COVID-19) pandemic has increased the gap^3^ between the need for and availability of rehabilitation services. During the pandemic, more than half (62%, 101 of 163) of surveyed countries had experienced disruption of rehabilitation services, possibly as a result of resources being diverted towards COVID-19 care,^4^^,^^5^ disruptions that jeopardized the quality of life and functioning of populations.

**Fig. 1:**
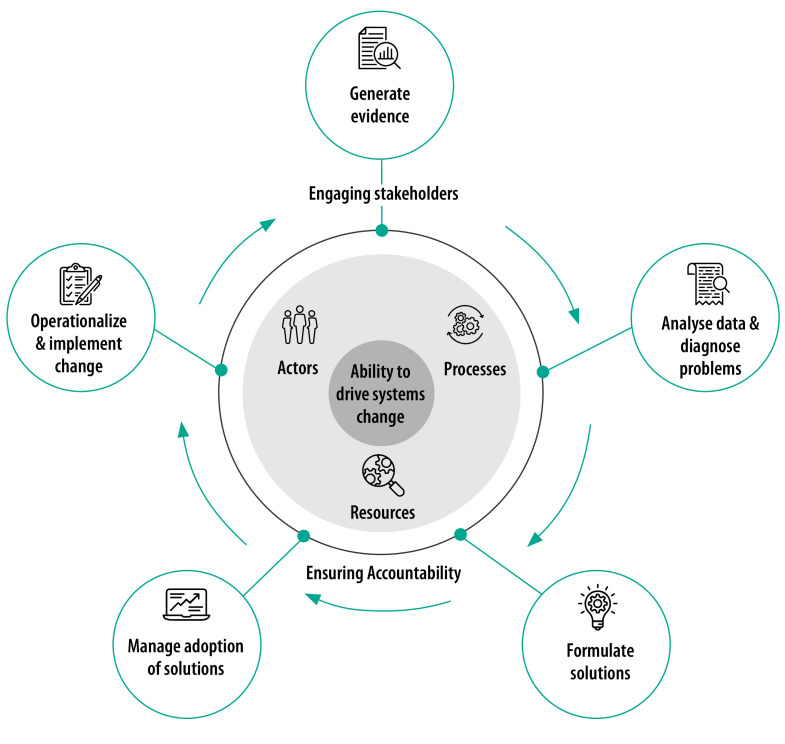
Institutional architecture for health system strengthening framework

Notes: Institutional architecture for health systems strengthening refers to the processes, actors and resources that interact – or fail to interact – to create health system improvements. The architecture includes activities to generate, analyse and use evidence to formulate health systems strengthening solutions, manage their adoption and implement the change. The framework helps countries to self-assess essential capacities to perform key health systems strengthening functions, plan for building capacities and track improvements. For this article, we adapted this framework to map processes, actors and resources that are necessary to implement selected policies for strategic health purchasing and perform a combination of functions outlined in the framework. Policy-makers can use this mapping to understand the stakeholder landscape and resource requirements of strategic purchasing initiatives.

Adequate health financing is important in ensuring efficient, integrated and sustainable delivery of rehabilitation services.^6^^,^^7^ Such financing mobilizes revenue for health services, including for rehabilitation, and makes the best use of resources by pooling these funds and being strategic about purchasing services on behalf of the population.^8^ In this article, we discuss why strategic purchasing in resource-constrained settings can expand coverage of rehabilitation services, and introduce three related policy priorities. Adapting a framework that describes how countries drive health system improvement (Fig. 1),^9^ we outline the key processes, actors and resources that are needed to implement the three policy priorities.

This article is directed towards policy-makers in middle-income countries, and includes experiences from high-income countries.

## Strategic purchasing

Strategic purchasing can improve service outcomes and promote integration of rehabilitation services into health systems. Purchasing refers to the transfer of funds from third-party payers to health-care providers to pay for services delivered to a covered population.^10^ Strategic purchasing refers to deliberate efforts to link these payments with population health needs or service provider performance. Such purchasing uses policy instruments such as clearly defined benefits packages, contracting and provider payment mechanisms to maximize equitable and quality service outcomes from constrained resources.^11^^,^^12^ For rehabilitation, strategic purchasing approaches consider: (i) What does the population need in relation to rehabilitation? Which health conditions can affect people’s functioning and create the largest disability burden?; (ii) Which rehabilitation services are available at different levels of health care (i.e. primary, secondary, tertiary, specialized) and can be financed to effectively meet this need?; (iii) Which public and private providers can best deliver these services from specialized rehabilitation institutions, rehabilitation wards in facilities, individual rehabilitation providers or assistive technology providers, including pharmacies?; and (iv) How to standardize and set tariffs, payment models and quality measures for rehabilitation services, considering the diversity of need among individual users and the diversity of providers delivering rehabilitation care.

Through these considerations, strategic purchasing focuses resources on outcomes that expand financial protection and rehabilitation service coverage and help countries progress towards universal health coverage goals.^13^

Strategic purchasing is technically intricate, capacity intensive and politically complex to implement.^14^^,^^15^ The peculiarities of financing and delivery of rehabilitation services add layers of complexity. For example, there is a general lack of coordination and accountability across the various sectors financing, purchasing and delivering rehabilitation services; the data needed to drive strategic purchasing decisions are not available, of poor quality or insufficient to inform true service needs and track service outcomes;^16^^,^^17^ and outcomes themselves are difficult to define and standardize in health care settings as they are measured by indicators of functionality and can vary according to the treatment goals and subjective experience of individual patients.^6^^,^^18^^,^^19^ Significant gaps in rehabilitation service availability^20^ and access can create additional hurdles for purchasers trying to link payment to quality or equity outcomes.

Despite these complexities, rehabilitation purchasers can rely on several policy options and instruments to improve purchasing decisions.^21^

## Policy priorities

The transition from passive purchasing to strategic purchasing represents a change in thinking in how rehabilitation services are prioritized, paid for and delivered. We present three key policy priorities that can promote this shift and enable reforms to improve efficiency and quality of care: (i) developing and prioritizing evidence-based benefits packages for rehabilitation; (ii) using contracting and provider payment mechanisms to pay for quality and efficient rehabilitation services; and (iii) instituting and strengthening stewardship for purchasing rehabilitation services.

These policy priorities, their implementation components and country examples are based on consultations with health financing and rehabilitation experts and peer-to-peer exchanges with practitioners from over 40 countries.^17^ The processes, actors and resources needed to implement the priorities are also informed by experiences of strategic health purchasing reforms in other health service areas.

### Evidence-based benefits packages

A national health benefits package refers to the set of services that can be feasibly financed and provided within a country’s context.^22^ Strategic purchasers should explicitly define priority rehabilitation interventions to be included in a health benefits package and align them with population needs, service provision capacities and available resources.^23^ Table 1 lists key processes, actors and resources that are integral to developing and prioritizing benefits packages for rehabilitation within existing health financing systems.

**Table 1:** Processes, actors and resources for developing and prioritizing evidence-based benefits packages for rehabilitation

Processes	Actors	Resources
• Estimating population service needs, demand and supply for rehabilitation • Including rehabilitation stakeholders in national priority-setting processes for the health sector • Convening multisectoral working groups to prioritize rehabilitation interventions in health financing mechanisms • Specifying the types and mix of interventions to offer in benefits packages • Setting tariffs and service delivery standards for these interventions • Developing referral and gatekeeping pathways for rehabilitation services • Ensuring the population understand the benefits • Establishing mechanisms for routine updates to benefits packages	• National or subnational health systems stewards (if different from purchasers) • Purchasers • Providers • Professional associations, academia and researchers • Civil society, especially disabled people’s organizations, and other service user groups • External donors • Other policy-makers	• Data on burden of diseases that may need rehabilitation services, disability prevalence, and service availability, readiness and cost • Health technology assessments for rehabilitation interventions • Budget impact assessments • Communication platforms with users and providers

Rehabilitation service packages should be developed within the existing health service prioritization framework. Essential processes include estimating population need, comparing need to currently available services and potential solutions, estimating related costs, setting tariffs and matching the package with available financial resources. This approach will help avoid vague, non-transparent and underfunded benefits packages that are occasionally observed in publicly funded systems in low- and middle-income countries.^22^ For example, the Explicit Health Guarantees package^24^ of Chile covers rehabilitation services in response to the 2016–2017 National Health Survey, which estimated that 42% of the Chilean population had moderately or severely reduced functioning that might benefit from rehabilitation.^25^ According to the country’s existing prioritization framework, inclusion in this list depends on the severity and frequency of the health condition the service addresses, the intervention’s effectiveness, service delivery capacities and available funding. Selected rehabilitation services satisfied all these criteria before inclusion in the package.^25^

Rehabilitation benefits should be selected by multistakeholder committees or working groups, and based on prioritization frameworks. Because of the close linkage of rehabilitation to the rights of people living with disabilities, civil society actors such as organizations of people with disabilities can play a fundamental role in ensuring user responsiveness of benefits and advocating to prioritize rehabilitation in financing schemes. In the Philippines, such organizations helped facilitate the prioritization and the inclusion of select mobility, prosthetic, orthotic and rehabilitation services in the Z Benefits package under the national health insurance scheme,^26^ making the Philippines one of the exemplary countries to explicitly spell out the type and volume of rehabilitation services provided through a financing scheme.^17^

Data and evidence are essential resources for the formulation of benefits packages. Rehabilitation need is frequently informed by disability data, or prevalence of health conditions that can cause permanent or temporary functional difficulties. Looking beyond disability data is important in capturing rehabilitation needs of individuals without a disability status or with cognitive functional challenges that may not be adequately captured by self-reported surveys.^27^ Health technology assessment can help prioritize cost-effective services with the highest return on investment. Such assessments for rehabilitation services are scarce in middle-income countries, and when such country-level evidence is not available, policy-makers can refer to global guidance on rehabilitation needs estimation and cost–effectiveness of rehabilitation interventions.^28^ Data and research on rehabilitation service availability, readiness, costs and budget impact can inform what might be realistically planned and provided, and should ultimately drive prioritization processes. This approach promotes budget accountability and transparency, and helps ensure that user, provider and purchaser expectations are aligned.^12^

### Provider payment mechanisms 

Contracts are negotiated agreements between purchasers and providers. Provider payment mechanisms can be part of contracts and define how funds are transferred to providers. These mechanisms are key tools for a strategic purchaser to promote the efficiency and quality of rehabilitation services.^29^ Table 2 highlights the main processes, actors and resources needed to develop contracts and implement provider payment mechanisms for rehabilitation services.

**Table 2:** Processes, actors and resources for using contracting and provider payment mechanisms to pay for quality and efficient rehabilitation services

Processes	Actors	Resources
• Developing contracting templates and processes among purchasers and providers • Contracting public, private and specialized providers of rehabilitation services • Choosing provider payment mechanisms for outpatient and inpatient rehabilitation services • Defining quality outcomes for purchased rehabilitation services • Developing systems to track outcomes of rehabilitation services • Building provider and purchaser capacities for financial and quality management	• Purchasers • Providers • Regulatory bodies • Professional associations • Technical and academic experts from local and global health community	• Data on rehabilitation inputs and outcomes • Costing exercises for rehabilitation services • Health information systems for managing claims, finances, services, outcomes and other data to monitor quality of and expenditure for rehabilitation services purchased • Training to continuously build human resource capacity to monitor and report outcomes, submit and manage claims and manage budgets

When contracting for health services, purchasers should develop legal frameworks for collaboration, identify public and private rehabilitation providers to contract, construct an agreement about what will be purchased, determine how it will be purchased, set minimum required standards for facilities and expected outcomes, and develop systems to continuously monitor provider performance.^29^ The format of the agreement can vary between and within countries, and not all rehabilitation contracts will cover these components. For example, Rwanda has recently adopted accreditation standards for rehabilitation services, which are used to qualify a facility to receive funds from public purchasers and establish a non-competitive contract with them. Contracting with private rehabilitation facilities in Rwanda is more structured and competitive, and depends on the agreements set with public funders, social insurers and private insurers.^30^ The purchaser of Pakistan’s Sehat Sahulat Program for people with disabilities competitively contracts a facility based on eligibility criteria.^31^ The contract mandates the provision of a select set of services free of charge to programme beneficiaries and retroactively reimburses based on submitted bills.

One common observation in low- and middle-income countries is that rehabilitation contracts often lack specifications on the expected outcomes of care.^17^ Strategic purchasing shifts the focus of investment from service inputs or outputs (e.g. physiotherapist salary or number of physiotherapy visits), towards service outcomes. This practice is novel to many low- and middle-income countries, whereas high-income countries with advanced financing systems for rehabilitation use measures such as improvements in functioning, autonomous living, discharge to community, return to the workforce, quality of life, reduced depression and patient satisfaction to track rehabilitation service outcomes.^32^^–^^34^ Countries can adopt and adapt these measures for their own strategic purchasing objectives; they can also harness tools and resources from high-income settings that can help classify and systematize coding of rehabilitation data to inform payment models and service outcomes.^35^^,^^36^

Implementing contracts and provider payment mechanisms can be resource intensive as they rely heavily on well-defined outcomes of care, information systems and data, as well as the capacity to report and monitor. Purchasers must find a sustainable balance between overly standardized, inflexible and passive payment practices that do not consider service outcomes – such as the voucher system for Georgia’s State Programme for Social Rehabilitation and Childcare^37^ – and overly complicated payment mechanisms with bespoke reporting requirements and fragmented reporting, such as the United States’ Medicare and Medicaid programmes.^38^ Countries with advanced strategic purchasing approaches are more efficient in tailoring payment mechanisms to different settings where rehabilitation services are provided – outpatient and inpatient – and variability in the expected outcomes of care. For example, Swiss insurance and some Canadian workers’ compensation schemes pay for each outpatient rehabilitation service with a pre-defined cap on the number of visits per treatment cycle.^18^^,^^19^ The cap can be adjusted based on individual patient needs by a referring clinician or an insurer (a gatekeeper). Inpatient rehabilitation is paid for by an activity-based mechanism or Diagnosis Related Groups by Australian Medicare and Medicare in the United States, respectively.^38^^,^^39^ Both mechanisms consider variations in condition, resource needs and required length of care at different levels of functional loss.

The scarce evidence on the effectiveness of different contracting and provider payment mechanisms for rehabilitation is limited to high-income settings and remains unexplored for low- and middle-income settings. Therefore, piloting and testing innovative approaches, conducting implementation research and other adaptive learning methods is needed to better understand the feasibility and impact of different contracting approaches and provider payment mechanisms on rehabilitation outcomes. Countries can learn from their own and peer experiences on different purchasing approaches for rehabilitation and the global health community should actively invest in this exchange.

### Stewardship for purchasing

Effective stewardship for strategic purchasing requires leadership to develop policies, set clear objectives, align various actors to achieve those objectives, and enforce the regulatory environment for purchasing arrangements.^12^^,^^21^^,^^40^ See Table 3 for implementation components of this policy priority.

**Table 3:** Processes, actors and resources for instituting and strengthening stewardship for purchasing rehabilitation services

Processes	Actors	Resources
• Identifying and empowering rehabilitation champion in the health ministry • Developing, adopting and implementing national health and rehabilitation strategies and operational plans to define policy objectives for actors and guide their roles • Establishing and enhancing coordination mechanisms to ensure alignment of actions and incentives of stewards, purchasers and providers • Implementing legal and organizational frameworks and reforms to establish institutional actors with clear roles and mandates • Establishing and maintaining user feedback channels	• Rehabilitation champions in the health ministry • National and subnational health systems stewards • Purchasers (national ministries of health, labour and/or social affairs; subnational governments; insurance agencies managing private health insurance and disability, accident or workers’ compensation schemes) • Finance ministry • Civil society, especially disabled people’s organizations, and other service user groups • Service providers	• Policies or other legal frameworks for purchasing rehabilitation services • Terms of reference or charters for coordination platforms • Strategic and operational plans for health sector that include rehabilitation • Financial management and business plans at the service provider level • Defined annual budgets and more conducive budgeting practices (programme-based classification, chart of accounts for providers) for strategic purchasing

One effective strategy is to identify and empower rehabilitation focal points within health ministries to champion health sector stewardship for rehabilitation, including purchasing policies. Rehabilitation services are purchased from health and non-health sectors. The latter include disability-focused programmes in the social, labour, defence or education sectors, or insurance schemes that manage disability, accident and/or workers’ compensation schemes.^41^ Further, national and subnational governments may be responsible for programming and purchasing different levels of rehabilitation care, requiring intragovernmental coordination and collaboration. Thus, health sector stewards of rehabilitation services need to set up or strengthen multisectoral and multistakeholder coordination platforms, develop policies and frameworks that clearly define roles and responsibilities of different purchasing institutions and align their objectives towards common population-level goals.

For example, the Rehabilitation Services Unit of the Kenya Ministry of Health leads the multistakeholder Rehabilitative and Assistive Technology Interagency Coordinating Committee and oversees the rehabilitation technical working group, which shapes county rehabilitation agendas and advises county directors for health on rehabilitation financing reforms – including for strategic purchasing. These reforms are coordinated at the county level with stakeholders from the health and non-health sectors. Reforms are guided by the National Rehabilitation and Assistive Technology Strategy and other national policies.^42^ Similarly, in Brazil, municipal governments are responsible for primary health care under the country’s highly decentralized Unified Health System. As part of implementing the national policy on family health support centres, they have benefited from financial support and policy direction from the national health ministry (from 2008 to 2016) to increasingly integrate rehabilitation into primary health care and successfully expand the number of qualified rehabilitation professionals (e.g. physiotherapists, speech therapists, psychologists and occupational therapists) offering services to those experiencing functional difficulties.^43^

Numerous legal, operational and technical resources can support stewardship of rehabilitation purchasing. These resources include strategic or operational plans, terms of reference for coordination platforms, and legal or policy documents that clarify the roles of various institutions, empower health sector actors to be effective stewards for rehabilitation and enable purchasers to be strategic in their investments.

Global momentum created by the Rehabilitation 2030 agenda^44^ provides an opportunity to promote health ministry stewardship. Rehabilitation in Health Systems: Guide for Action^45^ offers tools and platforms for situation assessment, strategic planning and stakeholder engagement.^46^ Health ministry leaders in El Salvador, Georgia and Jordan, among others, have effectively used these resources to convene stakeholders, establish a mutual understanding of the pressing challenges, and collaboratively identify pathways towards strengthening rehabilitation in health systems. These processes can also help facilitate discourses on strategic purchasing.

## The way forward

Strategic purchasing is a powerful but technically demanding approach to promoting quality and equity of services in resource-constrained settings. We propose following certain first-order priorities for policy-makers and managers committed to improving purchasing decisions for rehabilitation.

Purchasers need data to make strategic decisions, so an immediate priority should be identification and investment in data that can inform benefits, contracts, provider payment mechanisms and accountability mechanisms. While countries build capacities and cultures of data production, transparency and exchange among rehabilitation providers and multisectoral purchasers, they can adapt global resources and information to their local and/or national context.

Strategic purchasing decisions for rehabilitation should be inclusive and multisectoral. Rehabilitation is purchased and provided by multiple sectors. Without clarity on roles and accountability frameworks, everyone and no one is simultaneously accountable. In line with global momentum to strengthen rehabilitation care through a health system lens,^47^ the health sector should take on the stewardship role, collaborating and coordinating with other sectors to align purchasing efforts with achieving population-level goals.

Technical and academic stakeholders within the global health community have a key role to play in facilitating learning about and informing strategic health purchasing policies for rehabilitation. There is still a lot to learn about the feasibility and effectiveness of strategic purchasing instruments such as benefits packages, contracts and provider payment mechanisms for rehabilitation in resource-constrained settings and emerging health systems. The global health community should focus on innovating on, piloting, testing and studying these approaches to fill the knowledge gap and enrich the global evidence base with experiences from low- and middle-income countries. Over time, this experience will inform global normative instruments and resources for countries to use across different regions, resource settings and health systems contexts.

## Conclusion

The need to reinforce strategic use of funds for rehabilitation is urgent – especially in resource-constrained settings and considering post-COVID-19 realities. We present policy priorities that can help health system leaders make strategic purchasing decisions and target investments in ways that accelerate progress towards universal health coverage. We also encourage the global health community to invest in applied research on purchasing rehabilitation services, so policy-makers can assess innovative approaches and leverage high quality evidence throughout this journey.

## References

[1] Cieza A, Causey K, Kamenov K, Hanson SW, Chatterji S, Vos T. Global estimates of the need for rehabilitation based on the Global Burden of Disease study 2019: a systematic analysis for the Global Burden of Disease Study 2019. Lancet. 2020 Dec 19;396(10267):2006–17. 10.1016/S0140-6736(20)32340-0 33275908 PMC7811204

[2] Factsheet: Sustainable development goals: health targets: Rehabilitation. Copenhagen: World Health Organization Regional Office for Europe; 2019. Available from: https://apps.who.int/iris/bitstream/handle/10665/340896/WHO-EURO-2019-2384-42139-58051-eng.pdf [cited 2022 Jun 19].

[3] Cieza A. Rehabilitation the health strategy of the 21st century, really? Arch Phys Med Rehabil. 2019 Nov;100(11):2212–4. 10.1016/j.apmr.2019.05.019 31128114

[4] Haut ER, Leeds IL, Livingston DH. The effect on trauma care secondary to the COVID-19 pandemic: collateral damage from diversion of resources. Ann Surg. 2020 Sep 1;272(3):e204–7. 10.1097/SLA.0000000000004105 32452950 PMC7467027

[5] The impact of the COVID-19 pandemic on noncommunicable disease resources and services: results of a rapid assessment. Geneva: World Health Organization; 2020. Available from: https://www.who.int/publications/i/item/9789240010291 [cited 2022 Jun 19].

[6] Rehabilitation in health systems. Geneva: World Health Organization; 2017. Available from: https://www.ncbi.nlm.nih.gov/books/NBK552492/ [cited 2022 Mar 16].

[7] The world health report: health systems financing: the path to universal coverage: executive summary. Geneva: World Health Organization; 2010. Available from: https://apps.who.int/iris/handle/10665/70496 [cited 2022 Jun 19]. 10.2471/BLT.10.078741 PMC2878164 20539847

[8] Kutzin J. Health financing for universal coverage and health system performance: concepts and implications for policy. Bull World Health Organ. 2013 Aug 1;91(8):602–11. 10.2471/BLT.12.113985 23940408 PMC3738310

[9] Institutional architecture for health systems strengthening: summary report on a framework for application. Washington, DC: Results for Development; 2020. Available from: https://www.acceleratehss.org/wp-content/uploads/2021/02/HSSA-Summary-of-Institutional-Architecture-for-Health-Systems-Strengthening-May-2020.pdf [cited 2022 Jun 19].

[10] Mathauer I, Dale E, Jowett M, Kutzin J. Purchasing health services for universal health coverage: how to make it more strategic? Health financing policy brief no. 6. Geneva: World Health Organization; 2019. Available from: https://www.who.int/publications/i/item/WHO-UCH-HGF-PolicyBrief-19.6 [cited 2022 Jun 19].

[11] Gatome-Munyua A, Sieleunou I, Sory O, Cashin C. Why is strategic purchasing critical for universal health coverage in sub-Saharan Africa? Health Syst Reform. 2022 Mar 1;8(2):e2051795. 10.1080/23288604.2022.2051795 35446198

[12] Mathauer I, Dale E, Meessen B. Strategic purchasing for universal health coverage: key policy issues and questions: a summary from expert and practitioners’ discussions. Geneva: World Health Organization; 2017. Available from: https://apps.who.int/iris/handle/10665/259423 [cited 2022 Jun 19].

[13] Chikhradze T. Setting the scene for webinar 2. In: Rehabilitation in health financing: regional webinar series, Europe and Eastern Mediterranean, webinar 2; 2021 Sep 8; virtual [internet]. Health Systems Strengthening Accelerator; 2021. Available from: https://drive.google.com/file/d/1bqhnATFkXIBrX82za3WsR84p3uG6foMq/view [cited 2022 Jun 19].

[14] Greer SL, Klasa K, VAN Ginneken E. Power and purchasing: why strategic purchasing fails. Milbank Q. 2020 Sep;98(3):975–1020. 10.1111/1468-0009.12471 32749005 PMC7482378

[15] Hanson K, Barasa E, Honda A, Panichkriangkrai W, Patcharanarumol W. Strategic purchasing: the neglected health financing function for pursuing universal health coverage in low- and middle-income countries; Comment on “What’s needed to develop strategic purchasing in healthcare? Policy Lessons from a Realist Review”. Int J Health Policy Manag. 2019 Aug 1;8(8):501–4. 10.15171/ijhpm.2019.34 31441291 PMC6706967

[16] McPherson A, Durham J, Richards N, Gouda H, Rampatige R, Whittaker M. Strengthening health information systems for disability-related rehabilitation in LMICs. Health Policy Plan. 2016 Apr 1;32(3):384–94. 10.1093/heapol/czw140 27935799 PMC5400158

[17] Alperson R, Brainerd E, Chikhradze T, Ishtiaq A. Rehabilitation in health financing: final webinar report. Washington, DC: Health Systems Strengthening Accelerator, Results for Development; 2022. Available from: https://www.acceleratehss.org/wp-content/uploads/2022/03/Final-Report_Rehab-in-Health-Financing-Webinars_final_3.10.2022.pdf [cited 2022 Jun 19].

[18] Hudon A, Hunt M, Ehrmann Feldman D. Physiotherapy for injured workers in Canada: are insurers’ and clinics’ policies threatening good quality and equity of care? Results of a qualitative study. BMC Health Serv Res. 2018 Sep 3;18(1):682. 10.1186/s12913-018-3491-1 30176873 PMC6122715

[19] Halfon P, Eggli Y, Morel Y, Taffé P. The effect of patient, provider and financing regulations on the intensity of ambulatory physical therapy episodes: a multilevel analysis based on routinely available data. BMC Health Serv Res. 2015 Feb 7;15(1):52. 10.1186/s12913-015-0686-6 25889368 PMC4325958

[20] Bright T, Wallace S, Kuper H. A systematic review of access to rehabilitation for people with disabilities in low- and middle-income countries. Int J Environ Res Public Health. 2018 Oct 2;15(10):2165. 10.3390/ijerph15102165 30279358 PMC6210163

[21] Sanderson J, Lonsdale C, Mannion R. What’s needed to develop strategic purchasing in healthcare? Policy lessons from a realist review. Int J Health Policy Manag. 2019 Jan 1;8(1):4–17. 10.15171/ijhpm.2018.93 30709098 PMC6358649

[22] Glassman A, Giedion U, Sakuma Y, Smith PC. Defining a health benefits package: what are the necessary processes? Health Syst Reform. 2016 Jan 2;2(1):39–50. 10.1080/23288604.2016.1124171 31514661

[23] Glassman A, Giedion U, Smith PC. What’s in, what’s out: designing benefits for universal health coverage–key messages for donors and advocates. [version 1; not peer reviewed]. F1000 Res. 2017;6:1864. 10.7490/f1000research.1114963.1

[24] AUGE 85. Listado específico de prestaciones [internet]. Santiago: Ministerio de Salud, Gobierno de Chile; 2022. Spanish. Available from: https://auge.minsal.cl/problemasdesalud/lep [cited 2022 Sep 20].

[25] Zapata XN. Paquetes de servicios para la compra estratégica de servicios de rehabilitación. In: Rehabilitation in health financing: regional webinar series Americas, webinar 2; 2021 Sep 29; virtual. Health Systems Strengthening Accelerator; 2021. Spanish. Available from: https://drive.google.com/file/d/12t5lFbtNamptteKqchVvazLsGot7PP5N/view [cited 2022 Jun 19].

[26] Domingo AF. Rehabilitation in health financing, strategic purchasing—benefits package design in the context of the Republic Act No. 11223 – the UHC Act. In: Rehabilitation in Health Financing: Regional Webinar Series Southeast Asia and Western Pacific, webinar 2; 2021 Sep 9 virtual. Health Systems Strengthening Accelerator; 2021. Available from: https://drive.google.com/file/d/1CZ4YTjtgdu9Lq8zfOovNif6EosIy9NPS/view [cited 2022 Jun 19].

[27] Mitra S. Making data and statistics more inclusive in developing countries. 2021 Apr 26. In: Let’s talk development. Washington, DC: World Bank Group; 2022. Available from: https://blogs.worldbank.org/developmenttalk/making-data-and-statistics-more-inclusive-developing-countries [cited 2022 Jun 19].

[28] Rauch A, Negrini S, Cieza A. Toward strengthening rehabilitation in health systems: methods used to develop a WHO package of rehabilitation interventions. Arch Phys Med Rehabil. 2019 Nov;100(11):2205–11. 10.1016/j.apmr.2019.06.002 31207218

[29] Figueras J, Robinson R, Jakubowski E. Purchasing to improve health systems performance. London: McGraw-Hill Education UK; 2005.

[30] Kayobotsi P. Contracting and provider payment mechanisms for strategic purchasing of quality rehabilitation care in Rwanda. In: Rehabilitation in health financing: regional webinar series Africa, webinar 2; 2021 Oct 7; virtual. Health Systems Strengthening Accelerator; 2021. Available from: https://drive.google.com/file/d/1lKnWTmG9EUeavOvdq-Cu055PUPorqtci/view [cited 2022 Jun 19].

[31] Afzal S. Strategic purchasing: contracting and provider payment mechanisms. In: Rehabilitation in health financing: regional webinar series Europe and Eastern Mediterranean, webinar 2; 2021 Sept 8; virtual. Health Systems Strengthening Accelerator; 2021. Available from: https://drive.google.com/file/d/1bqhnATFkXIBrX82za3WsR84p3uG6foMq/view [cited 2022 Jun 19].

[32] Walters R, Collier JM, Braighi Carvalho L, Langhorne P, Katijjahbe MA, Tan D, et al.; AVERT Trialists’ Collaboration. Exploring post acute rehabilitation service use and outcomes for working age stroke survivors (£65 years) in Australia, UK and South East Asia: data from the international AVERT trial. BMJ Open. 2020 Jun 11;10(6):e035850. 10.1136/bmjopen-2019-035850 32532772 PMC7295421

[33] Murray PK, Singer M, Dawson NV, Thomas CL, Cebul RD. Outcomes of rehabilitation services for nursing home residents. Arch Phys Med Rehabil. 2003 Aug;84(8):1129–36. 10.1016/S0003-9993(03)00149-7 12917850

[34] Cao YJ, Nie J, Noyes K. Inpatient rehabilitation service utilization and outcomes under US ACA Medicaid expansion. BMC Health Serv Res. 2021 Mar 20;21(1):258. 10.1186/s12913-021-06256-z 33743706 PMC7981887

[35] UK ROC: UK Rehabilitation Outcomes Collaborative [internet]. London: King’s College London; 2022. Available from: https://www.kcl.ac.uk/cicelysaunders/research/studies/uk-roc/index [cited 2022 Jun 19].

[36] GAS – Goal Attainment Scaling in Rehabilitation [internet]. London: King’s College London; 2022. Available from: https://www.kcl.ac.uk/cicelysaunders/resources/tools/gas [cited 2022 Jun 19].

[37] Situation assessment of rehabilitation in Georgia. Copenhagen: World Health Organization Regional Office for Europe; 2020. Available from: https://apps.who.int/iris/bitstream/handle/10665/341324/WHO-EURO-2021-2393-42148-58068-eng.pdf [cited 2022 Jun 19].

[38] Deutsch A. How to define and pay for rehabilitation services to achieve quality and efficiency? United States of America country presentation. In: Rehabilitation in health financing: regional webinar series Americas; 2021 Dec 2; virtual. Health Systems Strengthening Accelerator; 2021. Available from: https://drive.google.com/file/d/1o_ieVH6A2p6D0x7SY43JkVn3AOBpCoMy/view [cited 2022 Jun 19].

[39] Turner-Stokes L, Sutch S, Dredge R, Eagar K. International casemix and funding models: lessons for rehabilitation. Clin Rehabil. 2012 Mar;26(3):195–208. 10.1177/0269215511417468 22070989

[40] Everybody’s business: strengthening health systems to improve health outcomes. WHO’s framework for action. Geneva: World Health Organization; 2007. Available from: https://apps.who.int/iris/handle/10665/43918 [cited 2022 Jun 19].

[41] Ishtiaq A. Overview of activity & background. In: Rehabilitation in health financing: regional webinar series Europe and Eastern Mediterranean, webinar 1; 2021 Jul 14; virtual. Health Systems Strengthening Accelerator; 2021. Available from: https://drive.google.com/file/d/1zYwS4RoXQrY-Gw6djtZ8AH6tplZuMVMv/view [cited 2022 Jun 19].

[42] Tawa N. Coordination of different financing mechanisms to achieve equitable population coverage for rehabilitation services in Kenya. In: Rehabilitation in health financing: regional webinar series Africas, webinar 3; 2021 Nov 18; virtual. Health Systems Strengthening Accelerator; 2021. Available from: https://drive.google.com/file/d/1nEOzp0i70KA43L3XOLpP6w8WbvdX4vmr/view [cited 2022 Jun 19].

[43] da Silva DB, Dos Santos Sixel TR, de Almeida Medeiros A, Dos Santos Mota PH, Bousquat A, Schmitt ACB. The workforce for rehabilitation in primary health care in Brazil. Hum Resour Health. 2021 Oct 12;19(1):127. 10.1186/s12960-021-00669-x 34641877 PMC8507164

[44] Rehabilitation 2030 meeting report, Geneva: World Health Organization; 2019. Available from: https://cdn.who.int/media/docs/default-source/documents/health-topics/rehabilitation/meeting-report-rehab2030.pdf?sfvrsn=fedee563_5&download=true [cited 2022 Jun 19].

[45] Rehabilitation in health systems. Guide for action. Geneva: World Health Organization; 2019. Available from: https://www.who.int/publications/i/item/9789241515986 [cited 2022 Jun 19].

[46] Kleinitz P, Sabariego C, Cieza A. Development of the WHO STARS: a tool for the systematic assessment of rehabilitation situation. Arch Phys Med Rehabil. 2022 Jan;103(1):29–43. 10.1016/j.apmr.2021.04.025 34256023 PMC8769206

[47] Rehabilitation 2030: a call for action. Geneva: World Health Organization; 2017. Available from: https://apps.who.int/iris/bitstream/handle/10665/339910/9789240007208-eng.pdf?sequence=1&isAllowed=y [cited 2022 Jun 19].

